# The O-glycan is essential for the induction of protective antibodies against lethal infection by flagella A-bearing *Pseudomonas aeruginosa*

**DOI:** 10.1128/iai.00427-23

**Published:** 2024-02-23

**Authors:** Myeongjin Choi, Surekha Shridhar, Heather Fox, Kun Luo, Mohammed N. Amin, Sharon M. Tennant, Raphael Simon, Alan S. Cross

**Affiliations:** 1 Center for Vaccine Development and Global Health, University of Maryland School of Medicine, Baltimore, Maryland, USA; 2 Korea Institute of Toxicology, Daejeon, Republic of Korea; Stanford University School of Medicine, Stanford, California, USA

**Keywords:** *Pseudomonas aeruginosa*, flagellin, O-glycan, conjugate vaccine, *Klebsiella pneumoniae*, O polysaccharide

## Abstract

To address the problem of increased antimicrobial resistance, we developed a glycoconjugate vaccine comprised of O-polysaccharides (OPS) of the four most prevalent serotypes of *Klebsiella pneumoniae* (KP) linked to recombinant flagellin types A and B (rFlaA and rFlaB) of *Pseudomonas aeruginosa* (PA). Flagellin is the major subunit of the flagellar filament. Flagella A and B, essential virulence factors for PA, are glycosylated with different glycans. We previously reported that while both rFlaA and rFlaB were highly immunogenic, only the rFlaB antisera reduced PA motility and protected mice from lethal PA infection in a mouse model of thermal injury. Since recombinant flagellin is not glycosylated, we examined the possibility that the glycan on native FlaA (nFlaA) might be critical to functional immune responses. We compared the ability of nFlaA to that of native, deglycosylated FlaA (dnFlaA) to induce functionally active antisera. O glycan was removed from nFlaA with trifluoromethanesulfonic acid. Despite the similar high-titered anti-FlaA antibody levels elicited by nFlaA, rFlaA, and dnFlaA, only the nFlaA antisera inhibited PA motility and protected mice following lethal intraperitoneal bacterial challenge. Both the protective efficacy and carrier protein function of nFlaA were retained when conjugated to KP O1 OPS. We conclude that unlike the case with FlaB O glycan, the FlaA glycan is an important epitope for the induction of functionally active anti-FlaA antibodies.

## INTRODUCTION

*Klebsiella pneumoniae* (KP) and *Pseudomonas aeruginosa* (PA) are leading causes of both community-onset and healthcare-associated infections (1). With their dramatic increase in antimicrobial resistance and the collapse of the antibiotic development pipeline, the Centers for Disease Control and Prevention (CDC) has classified these pathogens as “urgent” and “serious” threats, respectively (2). Consequently, there has been a renewed interest in the development of vaccines for the prevention of these infections which might also reduce the need for antibiotics as well as decrease their transmissibility. The O-polysaccharides (OPS) of both of these bacterial genera have been shown to be targets for vaccine development (3, 4). However, since the T cell-independent polysaccharide antigens are poor immunogens, they have been covalently linked to carrier proteins, which enables the recruitment of T cell help and improves polysaccharide immunogenicity (5). Many currently licensed bacterial polysaccharide vaccines are formulated as glycoconjugates.

A quadrivalent glycoconjugate vaccine was developed and targets both KP and PA infections by conjugation of four O-polysaccharides of KP to a PA-relevant protein, the flagellar (Fla) protein, which is an essential virulence factor of PA (4). The four KP O-polysaccharides were chemically linked to either recombinant FlaB (rFlaB) or recombinant FlaA (rFlaA), both recombinant proteins being non-glycosylated. Nearly 80% of KP infections are caused by the four O-polysaccharides (6, 7). Nearly 100% of invasive PA isolates express flagellar proteins A (having two subtypes, A1 and A2) or B. In a recent survey of 386 invasive PA isolates, 59% expressed either FlaA1 (28%) or FlaA2 (31%) (8).

The Fla proteins are excellent carriers for the KP OPS as demonstrated by the marked increase in anti-KP OPS antibodies when conjugated (4). In contrast, when simply admixed with the KP OPS (i.e., not conjugated), there was little KP OPS antibody formation. Significantly, PA flagellin is a potent TLR5 agonist that, when administered to human subjects, is highly reactogenic (9). However, there was a loss of TLR5 signaling when recombinant FlaA (rFlaA) or recombinant FlaB (rFlaB) underwent conjugation to the KP polysaccharides (4). The rFlaA and rFlaB proteins used in this vaccine elicited robust antibody responses, which in the case of rFlaB-induced antibodies were protective against experimental infection and reduced PA motility *in vitro*. However, while the rFlaA elicited a strong immune response, these antibodies lacked functional activity against FlaA-bearing PA strains (4). Since nearly 60% of invasive PA clinical isolates express FlaA, a successful PA flagellar vaccine must elicit functionally active antibodies to FlaA as well as to FlaB.

Further investigation of this result led to the evaluation of the glycosylation properties of the Fla proteins. Native FlaA and FlaB are glycosylated with different glycans, but neither rFlaA nor rFlaB, used as carrier proteins in the KP quadrivalent vaccine, are glycosylated (10, 11). We now report that whereas antisera to the rFlaA that lacks glycosylation are not protective in murine challenge studies, antisera to the fully glycosylated native FlaA (nFlaA) are highly protective when given either alone or as part of a glycoconjugate vaccine. These findings suggest that unlike the case for FlaB, the FlaA glycan is part of the protective epitope and should be conserved.

## MATERIALS AND METHODS

### Purification of native flagellin FlaA

Flagella were prepared as previously described (12). Briefly, *Pseudomonas aeruginosa* strain PAK (IATS O6, FlaA1) was grown in 2 L of Hy-Soy media [5 g/L sodium chloride (MilliporeSigma, MA), 10 g/L soytone (Teknova, CA), 5 g/L Hy-yest (Kerry BioScience, WI)] at 37°C and 80 rpm for 24 h. Bacteria were harvested by pelleting the culture at 10,900 × *g* at 4°C for 20 min. The pellet was resuspended in 40 mL of cold phosphate buffered saline (PBS) at pH 7.4. Flagella filaments were sheared by blending in a Waring blender for 2 min at 4°C. Sheared flagellin filaments were pelleted by ultracentrifugation at 100,000 *× g* at 4°C for 4 h and dissolved in PBS at pH 7.4. Flagellin filaments were monomerized by lowering the pH to 2.0 by adding 5 M HCl and stirring at room temperature for 30 min. Purified nFlaA was collected by final ultracentrifugation at 100,000 *× g* at 4°C for 4 h. The pH was increased to 7.0 and sterile filtered with 0.22-µm filter (Millipore, MA) and stored at −20°C. Purified nFlaA was analyzed by 4%–20% Tris-Glycine SDS-PAGE (Invitrogen) with Coomassie staining and Western blot with mouse anti-FlaA antibody. The protein concentration was determined by the bicinchoninic acid assay (BCA) method. Endotoxin levels were assessed with the Endosafe PTS and nexgen-PTS systems with the use of Endosafe PTS chromogenic Limulus amebocyte lysate assay cartridges (Charles River, MA). Lipopolysaccharide (LPS) contaminants from purified nFlaA were removed by batch binding with polymyxin B resin (Sigma, MA). Pure nFlaA devoid of LPS was eluted in PBS at pH 7.4. Protein-containing fractions were confirmed by SDS-PAGE with Coomassie blue staining. Endotoxin levels were assessed as described above.

### Chemical deglycosylation of flagellin

In order to study the role of the glycans in flagellar functional activity, we sought to remove the O-linked glycans. While PNGaseF enzymatically removes N-linked glycans from proteins, until recently, no such enzymes have been available to remove the O-linked glycans. We tried, unsuccessfully, to remove the O glycans with Ogly-ZORR (Genovis, Inc., MA), an enzyme that removes mucin-type core 1 O glycans (see Fig. S1). Consequently, we employed chemical deglycosylation.

Purified flagellin was desalted by dialysis in milli-Q (Millipore) water, thoroughly dried by lyophilization, and transferred to glass vials. Deglycosylation was carried out by using the GlycoFree Chemical Deglycosylation kit according to the manufacturer’s protocol (PROzyme). Briefly, 50 µL of trifluoromethanesulfonic (TFMS) acid/toluene mixture was added slowly to the protein samples in each glass vial placed in a dry ice-ethanol bath and incubated at −20°C for 4 h. After 4 h, 150 µL of pyridine solution (pyridine/methanol/water in a 3:1:1 ratio) was added to each glass vial placed in a dry ice-ethanol bath for 5 min and transferred to wet ice for a further 15 min. The reaction mixture was neutralized by adding 400 µL of neutralization solution [0.5% (wt/vol) ammonium bicarbonate] and mixed briefly. Deglycosylated protein was recovered by centrifugation. Briefly, the sample was cooled to 4°C for 30 min and centrifuged at high speed 17,020 × *g* for 15 min. Pelleted protein was dissolved in 8 M urea and step-dialyzed in PBS buffer containing decreasing concentration of urea (6 M, 4 M, 2 M, and 0 M) using Slide-A-Lyzer dialysis cassette with 10,000-Da MWCO (Thermo Scientific). The sample was electrophoresed on 4%–25% Tris-Glycine SDS-PAGE with Coomassie staining to assess the deglycosylation. The final protein concentration was determined by BCA protein assay. Endotoxin levels were determined as described above.

### Preparation of nFlaA/KP OPS conjugate vaccine

KP O1 polysaccharide was purified and prepared for conjugation from strain CVD 3001 as previously described (4). Native FlaA was purified as described above and labeled with sulfo-GMBS. Protein labeling and the remaining conjugation steps were performed as previously detailed (4). Specifically, labeled nFlaA was purified and diafiltered into appropriate buffer with a 10-kDa tangential flow filtration membrane. The purified GMBS-nFlaA was conjugated to the labeled OPS in a ratio of 6:1 wt:wt of OPS to nFlaA and subsequently purified over a Superdex 200 16/600 column (Sigma, MA) run on an AKTA chromatography system. The polysaccharide and protein content in the purified conjugates were assessed by resorcinol and BCA assays with polysaccharide and unconjugated protein standards, respectively. Residual endotoxin was assessed by Limulus amebocyte lysate assay as described above. Size was evaluated by ultra-performance liquid chromatography with size exclusion chromatography (UPLC-SEC) with a BioZen 1.8-µm SEC-3 column (Phenomenex, CA) run at 0.3 mL/min on an Acquity H-Class Plus Bio System (Waters, MA) in PBS with 0.02% sodium azide, pH 7.4, and detection by UV280.

### Enzyme-linked immunosorbent assay (ELISA)

ELISA was used to measure serum IgG levels before and after immunization. For anti-FlaA titration, clear flat-bottom Microlon medium binding plates (Greiner Bio-One, NC) were coated with 2 µg/mL of FlaA in 0.05 M sodium carbonate buffer, pH 9.6, for 3 h at 37°C. For PA O6 COPS (core and O-polysaccharide) titration, clear flat-bottom Microlon medium binding plates (Greiner Bio-One) were coated with 10 µg/mL of PAK (IATS O6, FlaA1) COPS in sodium carbonate buffer, pH 9.6, for 3 h at 37°C. For *P. aeruginosa* strain IATS O2/16 COPS ELISA, plates were coated with either 10 µg/mL of O2/16 COPS or 10 µg/mL of *P. aeruginosa* strain (IATS O2/16) crude LPS lysate. Following coating, the plates were washed with PBS, pH 7.4, + 0.05% Tween-20 (PBS-T) six times with 2-min soak in between. The plates were blocked with 10% non-fat dry milk Omniblok (American Bio) in PBS, pH 7.4, at 4°C overnight. After washing the plates as described above, mouse serum samples were diluted in PBS-T + 10% non-fat dry milk Omniblok, added in duplicate to the plates, and incubated for 1 h at 37°C. Following washes, the bound mouse IgG was detected by horseradish peroxidase (HRP)-labeled Goat anti-mouse IgG (Invitrogen) diluted to 1:2,000 in PBS-T + 10% non-fat dry milk at 37°C for 1 h. After washes, substrate 3,3′,5,5′-tetramethylbenzidine (Thermo Scientific) was added and incubated at ambient temperature for 15 min in darkness. The reaction was stopped by adding 2 N sulfuric acid (Macron Fine Chemicals, PA), and the absorbance at 450 nm was recorded using Spectra Max Plus reader (Molecular Devices, CA) and SoftMax Pro software. End point titers were reported as ELISA units, which represents the absorbance multiplied by serum dilution just above 0.2 OD_450_.

### Motility inhibition assay

*P. aeruginosa* strain PAK was grown overnight at 37°C without shaking, in Hy-Soy medium to stationary phase. The cells were pelleted at 2,602 × *g* for 20 min at 4°C, washed twice with PBS pH 7.4, resuspended in PBS, and normalized to an OD_600_ of 1.0. The bacterial suspension was then diluted 1:1,000 in PBS. Semi-solid (soft) agar (1% tryptone, 0.5% NaCl, and 0.3% agar) was autoclaved for 20 min, at 121^o^C, cooled to 56°C for 30 min, and then at room temperature for 10 min. One milliliter of cooled agar was poured into each well of a 24-well plate containing pre-immune or post-immune sera yielding a final serum dilution of 1:30. Control wells contained no sera. After the agar set for 40 min at room temperature, the diluted *P. aeruginosa* cells were stabbed to the center of the agar well using a sterile toothpick and incubated at 30°C for 19 h in a humidified chamber. The motility halo originating from the center of inoculation was captured with a ChemiDocMP system (Bio-Rad Laboratories, CA), and the diameter of the motility was measured using ImageJ software (13).

### Toll-like receptor (TLR) 4 and 5 activity assays

TLR4 and TLR5 activity assays were performed as described previously with minor modifications (14, 15). Briefly, HEK-Blue-hTLR4 cells and HEK-Blue-hTLR5 cells carrying a secreted embryonic alkaline phosphatase (SEAP) reporter construct were obtained from InvivoGen (CA). Cells were maintained in Dulbecco's Modified Eagle Medium (DMEM) supplemented with 10% fetal bovine serum (FBS) and 0.5% penicillin/streptomycin with and without 0.2% Normocin (InvivoGen, CA) for TLR5 cells and TLR4 cells at 37^°^C with 5% CO_2_, respectively. Monolayers of 1 × 10^5^ cells per well in a 96-well plate were incubated with media alone, PBS, and FlaA and FlaB proteins at different concentrations ranging from 10 pg/mL to 10 µg/mL for 24 h. *E. coli* lipopolysaccharide O111:B4 (List Biological Laboratories, Inc., CA) was added to HEK-Blue-hTLR4 cell as a positive control. Twenty microliters of cell supernatant were added to QuantiBlue substrate (InvivoGen, CA) according to the manufacturer’s instructions. SEAP activity was measured as optical density at 620 nm. Curve fitting was performed to estimate the half maximal effective concentration (EC_50_) using a dose-response curve of GraphPad Prism v6.0 (GraphPad Software, Inc., CA). A strong correlation was considered if the correlation coefficient *R* was greater than 0.9.

### Animal studies

#### Mice immunization

Six- to seven-week-old female Crl:CD-1 mice (Charles River Laboratories, MA) were immunized with FlaA and FlaB proteins by intramuscular administration. Briefly, mice were immunized with 5 µg of proteins or PBS (negative control) on days 0, 14, and 28 (4). Sera were obtained prior to the first dose and 14 days after the last dose and stored at −20°C.

#### *P. aeruginosa* burn wound infections

Mice were infected with two *P. aeruginosa* isolates expressing FlaA1 flagellin protein as described previously with minor modifications (16). Briefly, mice were clipped a day before the burn procedure. On the following day, they were anesthetized, and then, an ignited flame was induced on the shaved back for 10 seconds to allow a nonlethal thermal injury on 10% of the body surface. Mice were challenged with 100 µL of 4 × 10^6^ CFU PAK (O6, FlaA1 type) on the burned site subcutaneously. In order to rule out any possible protection against *P. aeruginosa* due to immune responses to contaminating LPS from the O6 PA strain used to prepare nFlaA, 6 × 10^5^ CFU of a non-O6 strain of *P. aeruginosa*, clinical isolate IATS O2/16, FlaA1 type, was used in this model. Mice were given 500 µL of 0.9% sodium chloride (Baxter, IL) for rehydration. Mortality was recorded for 7 days after the infection.

### Statistics

All statistical analyses were performed with GraphPad Prism v6.0. Data for non-parametric distributions were analyzed by one-way analysis of variance (ANOVA) with Tukey’s multiple-comparison test, a non-parametric paired two-tailed *t*-test, and Mann-Whitney *U* test. Survival analyses for Kaplan-Meier curves were conducted by the Mantel-Cox log-rank test. *P*-values <0.05 were considered as statistically significant.

## RESULTS

### Chemical deglycosylation of flagellin

**Fig 1:**
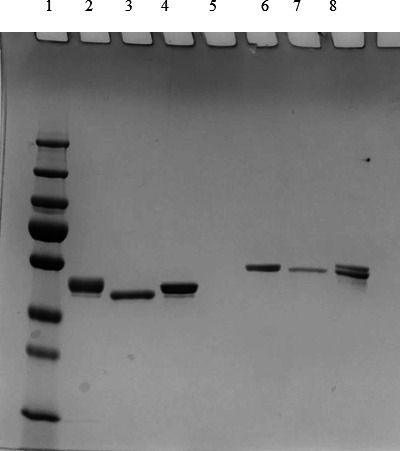
Chemical deglycosylation of native FlaA and native FlaB. Both native flagellin preparations were treated with TFMS as described in the Materials and Methods to remove the attached O-glycans. The preparations were then subjected to SDS-PAGE and stained with Coomassie blue. Lanes 1, protein molecular weight markers; 2, native FlaA; 3, deglycosylated FlaA; 4, recombinant FlaA-His tag; 5, empty; 6, native FlaB; 7, deglycosylated FlaB; and 8, recombinant FlaB-His tag.

In order to explain the differences between FlaA and FlaB in eliciting functionally active antibodies, it was hypothesized that the FlaA glycan may play an important role. Both FlaA and FlaB are O-glycosylated; however, the O-glycan on FlaA is considerably larger and more complex than the O-glycan on FlaB (10, 11, 17). Native FlaA has a complex O-glycan attached via rhamnose to two amino acid sites (tyrosine 189 and serine 260), whereas native FlaB has a simple glycan without rhamnose and is attached via deoxyhexosamine (10, 11, 17). While N-glycans are readily removed from proteins by the enzyme PNGaseF, enzymatic removal of O-glycans has not been possible. Consequently, we used TFMS acid, a “super acid,” to chemically remove the O-glycans (18). Native FlaA from *P. aeruginosa* strain PAK and native FlaB from *Pseudomonas* strain PAO1 (IATS O5 FlaB) were deglycosylated. After deglycosylation, the proteins were electrophoresed by SDS-PAGE and stained with Coomassie blue to evaluate the effect of deglycosylation on molecular weight (MW). Treatment of native FlaA with TFMS resulted in a decreased MW of nFlaA, but not of nFlaB (Fig. 1). This difference may reflect the fact that in contrast to the nFlaA, removal of the considerably smaller glycan from nFlaB might not have caused as great a shift in MW of FlaB.

#### Immune response in mice measured by ELISA

**Fig 2:**
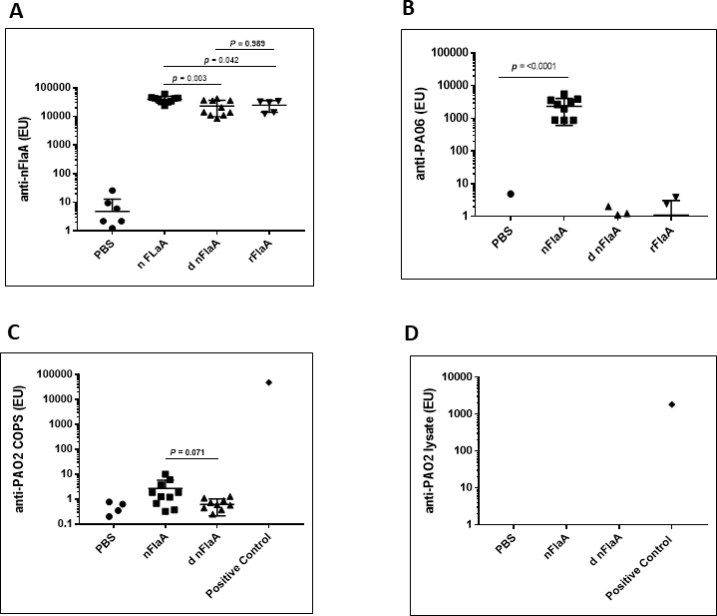
Immune response in mice to native FlaA (nFlaA), deglycosylated native FlaA (d nFlaA), recombinant FlaA (rFlaA), and placebo (PBS). Anti-nFlaA (panel A), anti-PAO6 (panel B), and anti-PAO2 titers were measured by ELISA. Anti-PAO2 titer was measured by either coating PAO2 COPS (panel C) or *P. aeruginosa* strain O2/16, proteinase-K-treated lysate (panel D). For positive control in the PAO2 COPS ELISA, sera from rabbits immunized with heat inactivated PA IATS PAO2 strain were used. ELISA units represent the absorbance multiplied by serum dilution just above 0.2. The differences among groups was analyzed by one-way ANOVA with Tukey’s multiple-comparison test. There was no difference in anti-nFlaA antibody titers among the three FlaA preparations.

Mice were immunized with nFlaA, deglycosylated nFlaA (dnFlaA), rFlaA, and PBS. Mice immunized with all three antigens generated very high anti-FlaA titers after three immunizations (Fig. 2A). Only mice immunized with native FlaA had high levels of anti-PAO6 COPS compared to deglycosylated native FlaA (Fig. 2B). This result is expected, as the endotoxin levels are high in native FlaA but decrease significantly after deglycosylation (Table 1). None of the mice had detectable anti-PAO2 levels when measured using either PAO2/16 COPS-coated or PAO2/16 lysate-coated ELISA plates (Fig. 2C and D). The FlaA+ PAO2/16 was a second challenge strain used to show the nFlaA conjugate vaccine protected against lethal PA infection.

**TABLE 1:** Endotoxin and beta-D-glucan levels

	Native FlaA	Deglycosylated FlaA	Recombinant FlaA
Endotoxin (EU/mL)	7,484	33.6	51.9
Glucan (pg/mL)	4,324	<100	N/A

#### Motility inhibition assay

**Fig 3:**
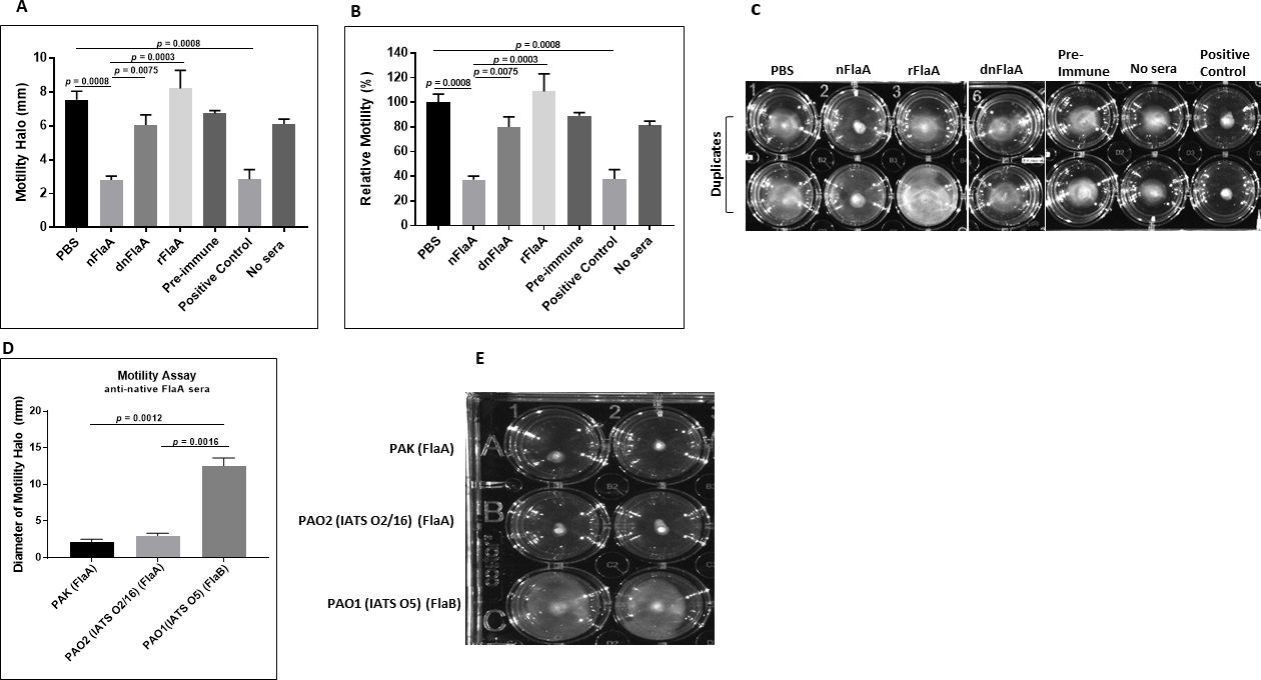
Motility inhibition of *P. aeruginosa* strain PAK (IATS O6 FlaA1). Pooled mice sera were assessed for the ability to inhibit motility of FlaA+ PA strain PAK, FlaA+ PA strain O2/16, and FlaB+ strain PAO1. Representative of four similar motility experiments. (**A and D**) The mean diameters (±SD) of the motility halos (in millimeters) in panels C and E were measured in duplicate (upper and lower rows) for each group. (**B**) Relative motility (i.e*.,* percentage of motility inhibition) from panel A was calculated after motility was normalized to that of the PBS group (pooled sera from mice immunized with PBS). (**C and E**) The diameter of the halo depicts the extent of the motility. The smaller the halo, the lesser the motility. The positive control used in this assay is anti-native FlaA sera. In the "No sera” control, the bacteria were added to the agar well containing PBS instead of sera. nFlaA, native FlaA; dnFlaA, deglycosylated native FlaA; rFlaA, recombinant FlaA. The differences among groups were analyzed by one-way ANOVA with Tukey’s multiple-comparison test.

Sera from mice immunized with either nFlaA, dnFlaA, or rFlaA were compared to determine the ability to inhibit the swimming motility of PA FlaA strain PAK. Sera from mice immunized with native FlaA (nFlaA) inhibited the motility of PAK (*P* < 0.0008 vs PBS and *P* < 0.0024 vs pre-immune sera) (Fig. 3). However, sera from the dnFlaA group did not inhibit PAK motility (NS vs PBS and pre-immune sera), suggesting that the deglycosylation might have altered a protective epitope of FlaA. As before, sera from rFlaA also did not inhibit PAK motility. The nFlaA antisera also inhibited the motility of another FlaA+ PA but not that of a FlaB-bearing PA (Fig. 3D and E).

### Endotoxin levels

Since antibodies to PA O-polysaccharides are protective in murine models of protection (3), before we tested the protective efficacy of antibodies to native FlaA, we assessed the endotoxin levels in the native FlaA preparations from which the FlaA was purified. The endotoxin level was drastically reduced after deglycosylation (Table 1). PA strains inherently have high levels of beta-glucan which can interfere with the endotoxin detection assay, giving false positives. We did indeed detect high levels of glucan in nFlaA samples, which could contribute to the high level of endotoxin observed in the native FlaA sample. The flagellar protein remained intact during the TFMS treatment, as evidenced by the protein migrating as a single band (Fig. 1).

### The presence of LPS in native flagellin was assessed by measuring SEAP secreted from hTLR4 reporter cells

**Fig 4:**
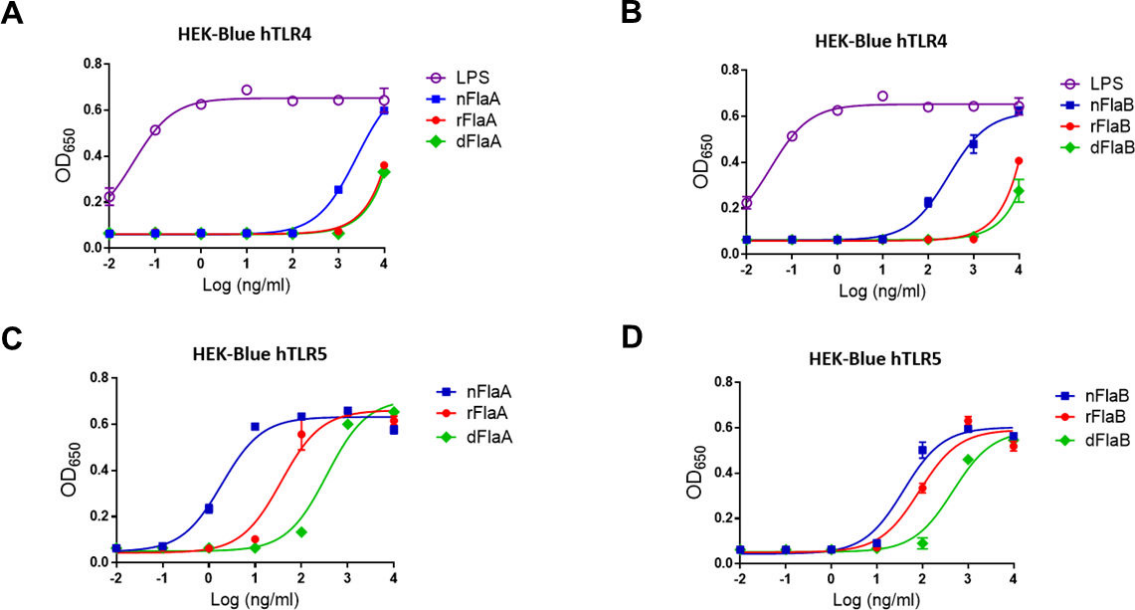
TLR4 and TLR5 bioactivity. *Pseudomonas* LPS, native flagellin (nFlaA and nFlaB), recombinant flagellin (rFlaA and rFlaB), and deglycosylated flagellin (dFlaA and dFlaB) were added to human TLR4 and TLR5 reporter cells. (**A**) FlaA formulations and LPS were added to human TLR4 reporter cells. (**B**) FlaB formulations and LPS were added to human TLR4 reporter cells. (**C**) FlaA formulations were added to human TLR5 reporter cells; (**D**) FlaB formulations were added to the TLR5 reporter cells.

Since impurified native flagellin with endotoxin were identified as described in Table 1, we used a TLR4 reporter assay to determine the comparative amount of LPS in each protein preparation. The mTLR4 cells were strongly responsive to LPS (purple circles) which is its agonist (Fig. 4A). The maximal TLR4 activation was achieved when cells were incubated with LPS and nFlaA, (blue squares) each at the highest concentration used in the assay (10 µg/mL). The EC_50_ values of LPS and nFlaA were 0.075 and 1,195 ng/mL, respectively. However, the EC_50_ values for rFlaA (red circles) and dnFlaA (green diamonds) proteins were not determined due to weak TLR4 activation. Similar to nFlaA, 10 µg/mL of nFlaB protein (blue squares) stimulated maximal TLR4 activation (Fig. 4B). The EC_50_ value of nFlaB was 295.5 ng/mL, while that of rFlaB (red circles) and dnFlaB (green diamonds) was not estimated due to poor TLR4 activity.

### Recombinant flagellin proteins stimulated TLR5 activation

In order to see whether flagellin proteins without glycan can be recognized by the immune system, we performed the TLR5 reporter assay with each of the native flagellin preparations (i.e., glycosylated form), recombinant (non-glycosylated form), and chemically deglycosylated proteins. Among FlaA proteins, nFlaA showed the highest potency in mTLR5 reporter cells (Fig. 4C). Compared with the estimated EC_50_ of nFlaA at 2.1 ng/mL, those of rFlaA and dnFlaA were higher. The EC_50_ values of rFlaA and dnFlaA proteins were estimated at 41.5 and 291.5 ng/mL, respectively. In Fig. 4D, nFlaB induced strong TLR5 activation with the EC_50_ value of 44.4 ng/mL. Unlike the 19.8-fold decreased potency of rFlaA compared with nFlaA, rFlaB showed 2.3-fold reduced potency of TLR5 activity (EC_50_ = 104.3). dnFlaA protein showed the highest EC_50_ value of 442.2 ng/mL with TLR5 activation.

### Mice immunized with native FlaA, but not rFlaA, were protected against PA

**Fig 5:**
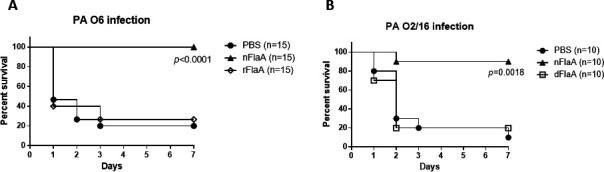
Immunization of mice with native FlaA (nFlaA) protects mice against lethal infection with FlaA-bearing strains of *Pseudomonas*. (**A**) Mice were immunized with either rFlaA or nFlaA prepared from a PAO6 strain (or given an equal volume of PBS) and challenged with PA O6 FlaA1. (**B**) Mice were immunized with either nFlaA or deglycosylated FlaA and challenged with FlaA-bearing *Pseudomonas* with a different IATS O type (PA O2/16) to ensure that the protection was not due to any residual LPS. (**A**) Immunization with nFlaA but not rFlaA protected the mice from lethal infection (*P* < 0.0001, Mantel-Cox log-rank test). (**B**) Immunization with nFlaA, but not with deglycosylated FlaA, protected against lethal infection with a FlaA-bearing strain but of a different O serotype (*P* < 0.0018, Mantel-Cox log-rank test).

To determine whether immunization with FlaA proteins without glycans would protect mice with burn injury against FlaA1-expressing *P. aeruginosa*, mice vaccinated with nFlaA and rFlaA were challenged with a sub-lethal dose of PAK (O6, FlaA1) and the PAO2/16, FlaA1 isolate; the latter is a FlaA^+^ PA strain of a different O serotype from which the nFlaA was prepared. Of 15 mice that received PBS, 20% of those who were infected with PAK survived (Fig. 5A). All the mice immunized with nFlaA survived, while only 26.7% of those immunized with rFlaA were alive after PAK infection (*P* < 0.0001, Mantel-Cox log-rank test). When mice with burn wound injury were infected with PAO2/16, the survival rate in non-immunized (i.e., PBS) mice was 10% (Fig. 5B). Interestingly, 90% of those immunized with nFlaA survived, whereas 20% of mice that received dFlaA were alive (*P* < 0.0018, Mantel-Cox log-rank test). In this rodent model, vaccination with nFlaA improved survival against two FlaA^+^ *P. aeruginosa* isolates significantly.

#### Preparation of a KP OPS/nFlaA conjugate vaccine

**Fig 6:**
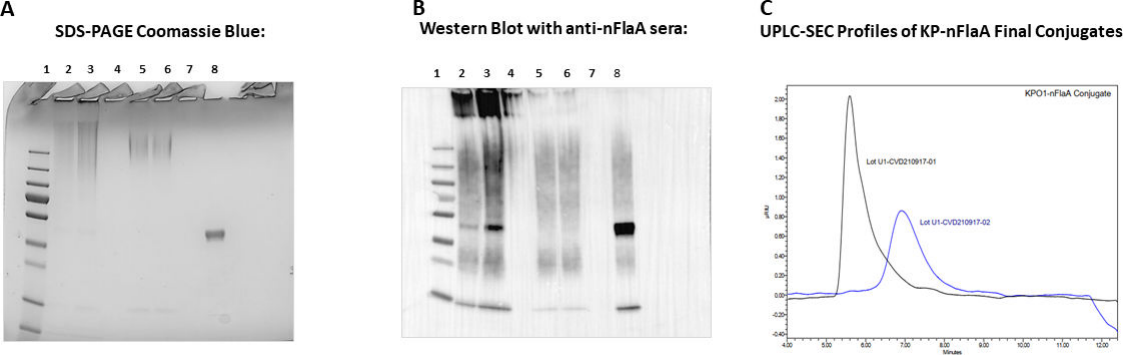
Conjugation of KP O polysaccharide to nFlaA. Two separate lots of nFlaA conjugate vaccines (conjugates 1 and 2) were prepared that differed in molecular weight as shown on SDS-PAGE (**A**), Western Blot (B), and size-exclusion chromatography (C). Lane 1, molecular weight marker; lane 2, KPO1-nFlaA conjugate 1 (2.5 μg); lane 3, KPO1-nFlaA conjugate 1 (5 μg); lane 4, empty; lane 5, KPO1-nFlaA conjugate 2 (1.13 μg); lane 6, KPO1-nFlaA conjugate 2 (1.13 μg); lane 7, empty; lane 8, nFlaA (1 µg). (**C**) UPLC-SEC profiles of KP-nFlaA final conjugates. Lot U1-CVD210917-01 (black trace) is higher molecular weight compared to lot U1-CVD210917-02 (blue trace). The SDS-PAGE gel shows high-molecular-weight conjugates with no free nFlaA. Anti-FlaA sera were used for the Western blot.

Based on the protective efficacy of nFlaA, a KP O1 OPS:nFlaA conjugate vaccine was prepared to determine if this formulation retained the ability to induce functionally active antibodies to FlaA-bearing PA following conjugation. It was observed after S200 purification using SDS-PAGE (Fig. 6, panel A) and Western blot (panel B) that there were two different sized conjugates present (Fig. 6, lanes 2 and 3 vs lanes 5 and 6). The S200 fractions were split into two separate lots. By size exclusion chromatography, the first lot had a slightly higher molecular weight compared to the second lot, which confirms the difference in sizes noted by SDS-PAGE (Fig. 6C). In the first lot, KPO1-nFlaA-01-01, the final conjugate had an nFlaA concentration of 0.16 mg/mL, an OPS:nFlaA ratio of 0.625:1, and endotoxin levels of 2.53 EU/mg OPS. In contrast, the second lot, KPO1-nFlaA-01-02, had an increased OPS:nFlaA ratio of 2.2:1, a lower nFlaA concentration of 0.05 mg/mL, and lower endotoxin level (Table 2).

**TABLE 2:** Data summary of KP-nFlaA final conjugates

Sample	Final nFlaA concentration, mg/mL (BCA)	OPS:nFlaA ratio	Endotoxin, EU/mg OPS
KPO1-nFlaA conjugate, lot U1-CVD210917-01	0.16	0.625:1	2.533
KPO1-nFlaA conjugate, lot U1-CVD210917-02	0.05	2.2:1	0.027

#### Immune response to the two KP O1 OPS/nFlaA vaccine lots

**Fig 7:**
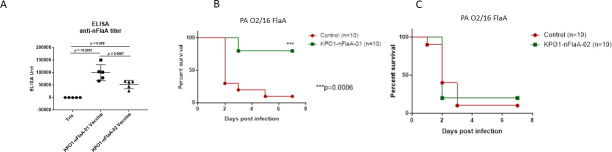
Differing protective efficacy in a mouse burn wound infection model of two batches of KP O1:nFlaA vaccines differing in nFlaA content. Eight- to ten-week-old CD-1 mice received three doses of either 10 µg KPO1-nFlaA-01 or KPO1-nFlaA-02 conjugate vaccine at 2-week intervals by subcutaneous administration. (**A**) KPO1-nFlaA conjugate O1 elicited a more robust IgG antibody response to FlaA than KPO1-nFlaA conjugate O2. (**B**) The protection of KPO1-nFlaA-Lot 01 conjugate vaccine in a mouse burn model. The mice were challenged with 6.4 × 10^5^ CFU of PA O2/16 (i.e., non-O6) immediately post-burn by subcutaneous administration. The survival rate of vaccine group was 80%, while the survival rate of control group was 10%. Using the Mantel-Cox log-rank test, the difference was significant (*P* = 0.0006). (**C**) The protection of KPO1-nFlaA-Lot 02 conjugate vaccine in a mouse burn model. The mice were challenged with 6.4 × 10^5^ CFU of PA O2/16 (i.e., non-O6) immediately post-burn by subcutaneous administration. The survival rate of the vaccine group was 20%, while the survival rate of control group was 10%.

Two groups of mice were immunized with the different lots of KPO1:nFlaA conjugate vaccine at days 0, 14, and 28 prior to subjecting them to burn wound infection (Fig. 7). While immunization with both lots of vaccine elicited a significant antibody response compared to those administered Tris, mice immunized with the 01 lot of conjugate vaccine had significantly higher anti-nFlaA antibody levels than those immunized with the 02 lot of vaccine (Fig. 7A; *P* < 0.01). Two weeks later, they were challenged with a non-O6 strain of PA and followed for survival. Eighty percent of mice that were immunized with the lot 01 conjugate (Fig. 7B) survived compared to 20% of mice immunized with the lot 02 vaccine (Fig. 7C) (*P* < 0.0230, Fisher exact test, two tailed). The difference in efficacy could be attributed either to the greater amounts of nFlaA in lot 01 or perhaps the shielding of the nFlaA by the greater amount of KP 01 OPS in lot O2.

**Fig 8:**
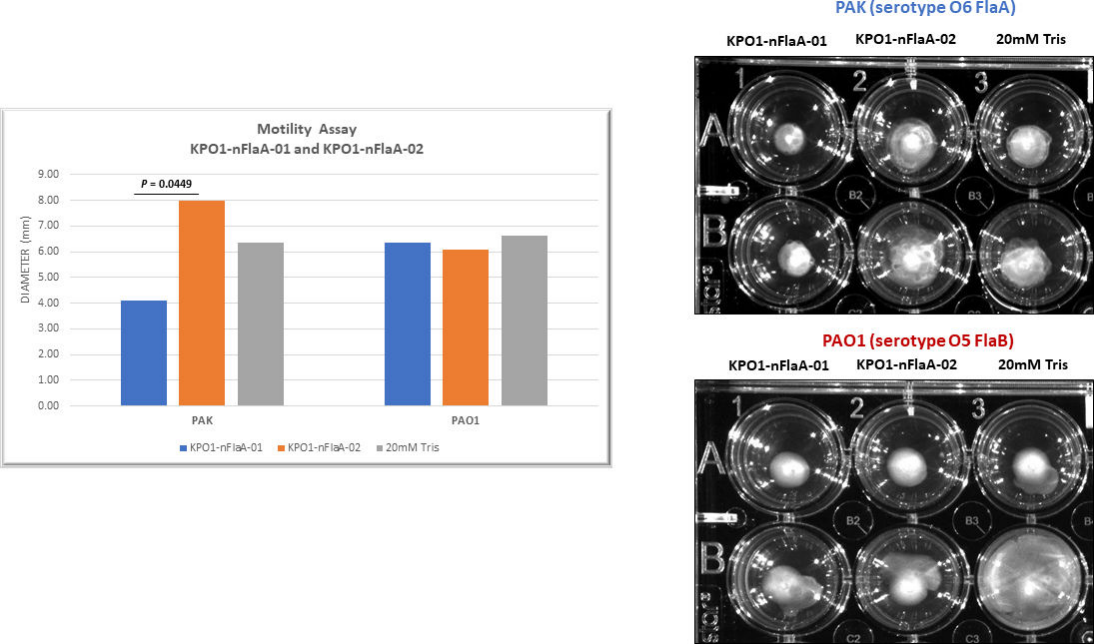
Antibodies elicited by KP O1-nFlaA-01 were better able to reduce the motility of a FlaA+ *Pseudomonas* strain than KP O1-nFlaA conjugate 2. Eight- to ten-week-old CD-1 mice received three doses of either 10 µg KPO1-nFlaA-01 or KPO1-nFlaA-02 conjugate vaccine at 2-week intervals by subcutaneous administration, and sera were harvested. The sera were diluted 1:50 and added to 0.3% soft tryptone agar and pre-incubated in a 24-well plate (in duplicate). *Pseudomonas* strains PAK (FlaA+) and PAO1 (FlaB+) were grown to log phase, normalized to OD 1.0, and diluted 1:1,000. Using a sterile toothpick, they were then stabbed centrally into the agar and incubated at 30°C with a wet towel. KPO1-nFlaA-01 sera inhibited the motility of PAK but not PAO1. KPO2-nFlaA-02 sera did not inhibit the motility of either PAK or PAO1. The differences between the PAK motility inhibition with antisera generated with the KP O1-nFlaA-01 and KP O1-nFlaA-O2 conjugate vaccines were analyzed with the non-parametric paired two-tailed *t*-test. There was no inhibition against the FlaB+ PAO1 strain.

**Fig 9:**
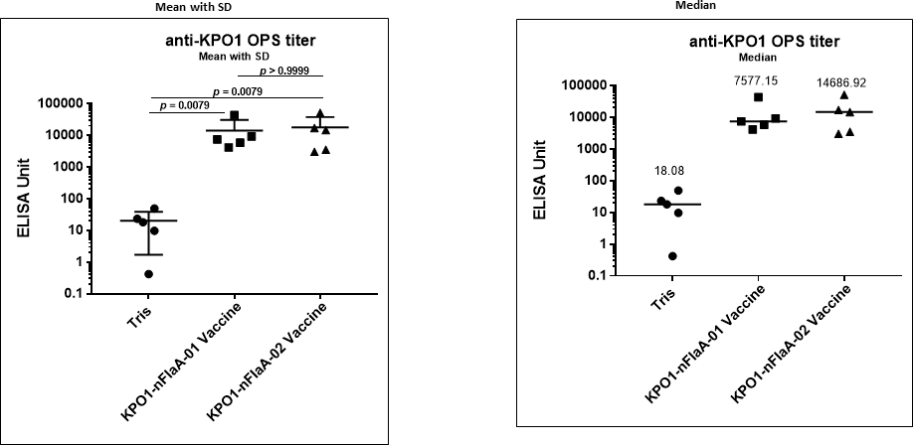
Native FlaA retains it carrier protein function when conjugated to KP O1 O-polysaccharide. Both KPO1-nFlaA conjugate vaccines 01 and 02 were able to enhance the immunogenicity of KP O1 OPS in mice following immunization subcutaneously with 10-µg OPS on days 0, 14, and 28. Sera were obtained at day 35 (7 days after the final vaccine dose), and KPO1 antibody levels were measured by ELISA. The differences between groups were analyzed by the Mann-Whitney *U* test.

The lot 01 conjugate was also better able to reduce the motility of a FlaA-bearing *Pseudomonas* (strain PAK) than the lot 02 conjugate (Fig. 8). Both lots of KP O1 OPS:nFlaA conjugates retained their carrier function in enhancing the anti-KP O1 OPS antibody response (Fig. 9).

## DISCUSSION

Our studies show that unlike the case for FlaB, the FlaA glycan is critical for the induction of functionally active antibodies against FlaA-bearing *Pseudomonas* strains. Furthermore, this epitope is retained when native FlaA is conjugated to the KP O-polysaccharide. Conjugation of flagella to the O-polysaccharide markedly reduced its TLR5 reactivity (4).

In a series of studies, the Ramphal laboratory had reported that PA FlaA and FlaB were differentially O-glycosylated (10, 11, 17). FlaA has a heterogeneous glycan comprising of up to 11 monosaccharide units O-linked to the protein through rhamnose residues on the flagellin backbone (10). The glycans on FlaA are localized to the central, surface-exposed domain of the monomer in the assembled filament. The TLR5 recognition sequences are localized in the conserved D1 domain of flagellin (18). In contrast, the O-linked glycan attached to the FlaB monomers at threonine and serine residues has fewer monosaccharides and is less heterogeneous than the glycan of FlaA (11). Our studies now show that these differences have different functional consequences with the O-glycan in FlaA being a critical functional antibody epitope.

In order to study the role of the glycans in flagellar functional activity, we had to remove the O-linked glycans. Since we were unsuccessful in removing the O glycans with Ogly-ZOR^R^ (Genovis, Inc.), we used the “superacid” TFMS to remove the glycan from native FlaA, as previously described (19). Surprisingly, such treatment had no observable effect on the FlaA protein backbone integrity (Fig. 1) while reducing endotoxin levels in the preparation.

There have been a great many PA vaccine candidates, including flagellin, but to date, there are none licensed (for review, see Doring and Pier) (20). PA flagellin has been proposed not only as a vaccine (21–23) but also as a carrier protein (24, 25) and adjuvant (26–28); however, most of these studies have used the native, glycosylated, not recombinant flagellin. PA flagellins are readily expressed and purified from heterologous Gram-negative bacterial expression systems, including *Salmonella* spp. and *Escherichia coli* (21). Campodononico and Pier reported that polymeric flagellin proteins (i.e., flagella) were superior to monomeric flagellin for generating an immune response to PA, but the flagellin monomer was a more potent activator of TLR5 activity than flagella. However, they concluded that the flagellar antigens alone would not induce solid immunity to PA (21).

Experimental studies with divalent, native (i.e., glycosylated) flagella preparations demonstrated flagella-specific protection independent of the O-antigen in murine burn injury models (29, 30) and later in respiratory infection models that in addition inhibited PA motility (31, 32). Clinical trials were conducted with bivalent PA flagella by ImmunoAG, first in 1971 (33) and then in 2007, with the latter being a phase III, double-blind, randomized, placebo-controlled study showing a decreased risk of infection in the 381 cystic fibrosis patients studied (22). These studies employed native flagellar proteins of subtype a_0_a_1_a_2_ from one *Pseudomonas* strain and subtype b from another. Consequently, the role of flagellar glycans in the induction of functional antibodies had not been directly addressed. Few reports, however, have used recombinant flagellar proteins as vaccines. Faezi and colleagues reported that immunization with rFlaA produced in *E. coli* provided 83% protection against burn wound infection with a FlaA-expressing PA and, surprisingly, protected 25% of mice infected with a FlaB-bearing PAs (34). The vaccine itself was not characterized in this study and specifically did not consider whether any LPS contamination may have contributed to the protection. PA flagella also induce a potent TLR5-signaling response that has limited their use as flagellar vaccines or as adjuvants (9).

Since the re-introduction of glycoconjugate vaccines for bacterial infections, many licensed vaccines have used a limited number of protein carriers, particularly tetanus and diphtheria toxoids. These proteins induce potent antibodies. However, as more glycoconjugate vaccines use a limited number of protein carriers, there is the danger of the carrier-induced antibodies causing carrier-induced suppression as we have seen in a previous study (3). Furthermore, the antibodies to these carrier proteins do not contribute to the host defenses against the bacteria to which the glycoconjugate vaccines are directed. In our KP O-polysaccharide/PA flagellar vaccine, we introduced the concept of “pathogen-relevant” carrier proteins (4), whereby the protein carrier chosen might also contribute to the protection against bacterial infection targeted by the vaccine. Since flagellin is an essential virulence factor for PAs and >95% of clinically relevant PA isolates carry either FlaA or FlaB, this KP OPS/PA flagellin vaccine targets two pathogens that are often resistant to multiple antimicrobial agents, which the CDC has labeled “urgent” and “serious” threats, respectively.

Similar to the role of FlaA-associated glycans in inducing a protective antibody response, Castric and colleagues described a second glycan-related pathway in PA which induces a protective antibody response (35, 36). They demonstrated that the glycosylated product of *PilA*, type IV pili, in PA strain 12.4.4 (PA IATS O8) induced protective antibodies against infection with the homologous O type from which the pili were isolated. Glycosylation of 12.4.4 pilin required the presence of the *pilO* gene, an oligosaccharide transferase that catalyzes O glycosylation of PA 12.4.4 pilin by adding a single O-antigen repeating unit to the C terminal residue. By showing structural similarity between the pilin glycan and the O-antigen of PA 12.4.4, they suggested that the pilin glycan of 12.4.4 is a product of the O-antigen biosynthetic pathway (37, 38). Moreover, they found that the O-antigen biosynthetic enzyme, WbpO, was able to use O-antigen genes from other PA O types or even from other Gram-negative bacteria which were then co-expressed on the individual pili with the O-antigens from the homologous PAK strain (38). The structural diversity of the O-antigens used by the 12.4.4 pilin glycosylation apparatus indicates that the glycan substrate specificity of the reaction is non-selective with regard to O-antigen structure. It even can add *E. coli* O-antigen. Since the pilin glycan stimulates a protective response that targets the O-antigen (39), they propose that this system may serve as the basis for a bioconjugate vaccine. These observations may explain the mechanism by which the passive administration of rabbit rPilA (type IV pili) IgG protected in a mouse burn wound model (40).

### Limitations

Our study has several limitations. PA has multiple virulence factors beyond flagellin. In addition to invasive strains such as PAK used here, PA strains express virulence factors that contribute to PA pathogenicity that are not affected by antibodies to flagella. For example, PA has a type 3 secretion system that uses PcrV as a syringe to deliver potent cytotoxins, such as Exo T and Exo U, to target cells and induce cellular injury. It also has genes that express polysaccharides that can form a biofilm to exclude antibiotics and immune factors as well as an LPS that is proinflammatory, although not as toxic on a weight basis as the LPS of *Enterobacteriacea*e. Furthermore, we also caution that because of strain differences, different PA strains may yield different results in our murine infection models. Another potential explanation for the role of the O-glycan of FlaA is the fact that, unlike FlaB, its glycan has a rhamnose. Anti-rhamnose antibodies are among the most abundant circulating natural antibodies (41). It has been proposed that antigens containing a rhamnose sugar may be recognized by the anti-rhamnose antibodies and be more efficiently presented to antigen-presenting cells through antigen uptake by the Fc receptors. If this were the case with the FlaA glycan, the antigen may be processed differently.

We conclude that despite the similar high-titered anti-FlaA antibody levels elicited by nFlaA, rFlaA, and dnFlaA, only the nFlaA antisera inhibited PA motility and protected mice following lethal intraperitoneal bacterial challenge. This suggests that the FlaA-associated glycan is critical to the induction of functionally active antibodies to FlaA. Based on our findings, we will modify our tetravalent *Klebsiella* OPS to be conjugated to recombinant FlaB and native FlaA flagellin. This glycoconjugate vaccine would cover 80% of circulating KP O types responsible for extra-intestinally invasive infections as well as >90% of invasive PA infections.

## AFTER EPUB

*[This article was published on 23 February 2024 with the middle initial missing for author Mohammed N. Amin. The byline was updated in the current version, posted on 27 February 2024.]*

## SUPPLEMENTAL MATERIAL

Figure S1 iai.00427-23-s0001.tif Enzymatic deglycosylation of native FlaA using OglyZOR enzymes. One unit each of the enzyme mix composed of the endoglycosidases SialEXO and OglyZOR enzyme was added per mcg of native FlaA in 20 mM Tris, pH 6.8. The reaction mixture was incubated for 4 hours at 37°C. The reaction mixture was run on SDS-PAGE and stained with Coomassie blue. Lane 1, protein molecular weight markers; lane 2, native FlaA before enzyme deglycosylation; lane 3, native FlaA after enzyme deglycosylation; lane 4, empty; lane 5, native FlaA after chemical deglycosylation.

ASM does not own the copyrights to Supplemental Material that may be linked to, or accessed through, an article. The authors have granted ASM a non-exclusive, world-wide license to publish the Supplemental Material files. Please contact the corresponding author directly for reuse.
