# Supplementary figures and images for: The O-glycan is essential for the induction of protective antibodies against lethal infection by flagella A-bearing Pseudomonas aeruginosa
Source: Infect Immun. 2024 Feb 23;92(3):e00427-23. doi: 10.1128/iai.00427-23 (PMC10929410; doi:10.1128/iai.00427-23)

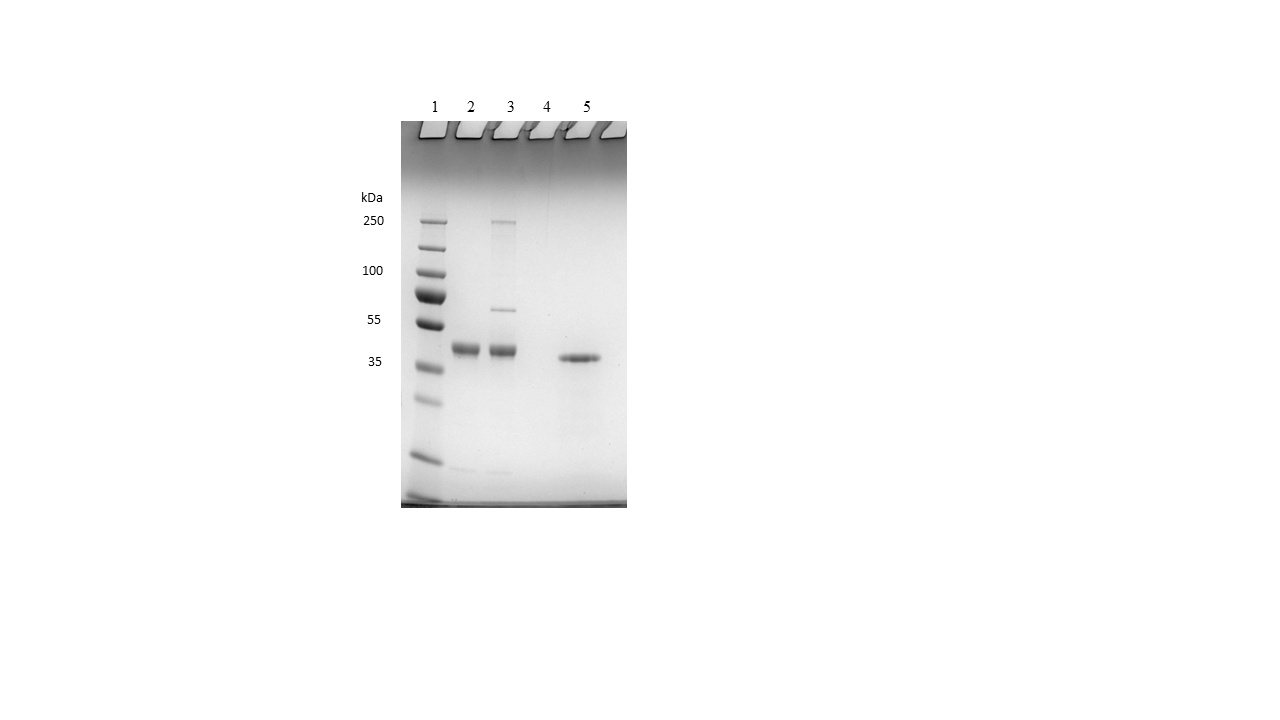

Supplement: Figure S1 — Enzymatic deglycosylation of native FlaA using OglyZOR enzymes. One unit each of the enzyme mix composed of the endoglycosidases SialEXO and OglyZOR enzyme was added per mcg of native FlaA in 20 mM Tris, pH 6.8. The reaction mixture was incubated for 4 hours at 37°C. The reaction mixture was run on SDS-PAGE and stained with Coomassie blue. Lane 1, protein molecular weight markers; lane 2, native FlaA before enzyme deglycosylation; lane 3, native FlaA after enzyme deglycosylation; lane 4, empty; lane 5, native FlaA after chemical deglycosylation. [file iai.00427-23-s0001.tif]
